# Application of the Health Belief Model (HBM) to Explore the Quality of Sexual and Reproductive Health (SRH) Education in Sri Lanka

**DOI:** 10.3390/ijerph21121703

**Published:** 2024-12-20

**Authors:** Wasantha Rajapakshe, Anjana Koushani Wickramasurendra, Rajini Ranmini Amarasinghe, Shynie Lourds Minoli Kohilawatta Arachchige Wijerathne, Nikini Devindi Wijesinghe, Naduni Madhavika

**Affiliations:** 1 SLIIT Business School, Sri Lanka Institute of Information Technology, Malabe 10110, Sri Lanka; 2 Tasmanian School of Business and Economics, University of Tasmania, Private Bag 84, Hobart 7001, Australia

**Keywords:** health belief model, ordered probit model, SRH education, Sri Lanka

## Abstract

Many countries, including Sri Lanka, are taking steps to integrate sex education into their educational systems to combat child abuse. However, this effort is often met with skepticism in Asian nations, including Sri Lanka. This study takes a unique approach by applying the criteria of the health belief model to predict the quality of reproductive health (SRH) education in Sri Lanka, offering a fresh perspective on this issue. A positive philosophical framework and a deductive approach have been employed to provide justification for the underlying assumptions. A structured questionnaire was used as the survey methodology, which included questions about external cues to action, self-efficacy, perceived barriers, perceived susceptibility, and perceived severity. Data was collected from a total of 384 Sri Lankan non-state undergraduate students to test their perception of these factors and how they affect the perceived benefits of quality SRH education. The level of self-efficacy, perceived susceptibility, and perceived severity yielded a coefficient estimate that was statistically significant, thus influencing the perceived benefits of quality SRH education. These results, obtained through a multivariate regression analysis, underscore the importance of one’s role in implementing effective SRH education. Importantly, there is no evidence that external cues to action and perceived barriers predict the perceived benefits of quality SRH education. This underscores the gravity of the situation and the need for immediate action. The findings of this study have significant practical implications. They can be used to develop an effective SRH program that aims to prevent sexual abuse among adolescents. This study also demonstrates that the health belief model can serve as a useful conceptual framework for such intervention programs, providing tangible solutions to the issue of SRH education quality.

## 1. Introduction

The risk of being raped among Sri Lankan youngsters is a grave concern, with the rate of rape in Sri Lanka showing a worrying trend. From 7.4 cases per 100,000 population in 2004 to 10.6 cases per 100,000 population in 2013, the rate of rape in Sri Lanka grew by 6.6% annually [1]. There were 1792 reported rape cases in Sri Lanka in 2018, and 1779 in 2019, with 1490 cases of rape against children reported in 2019 [2]. Shockingly, 14% of male and female youngsters in Sri Lanka under the age of 18 had experienced some form of sexual abuse [3]. From 2001 to 2018, sexual harassment was recorded against 84 children at a regional medical center in Sri Lanka [4]. The United Nations reports that the rape rate in Sri Lanka (11.3 cases per 100,000 population) is higher than that of several Asian countries, including India (9.10), Japan (5.6), Thailand (5.0), Pakistan (1.8), Myanmar (0.70), and the Philippines (1.8 cases per 100,000 population) [5]. Despite the severe impact it has on victims and their families, child sexual abuse has been mostly overlooked in Sri Lanka, highlighting the severity of the issue.

According to crime statistics published by the Sri Lanka Police Department, there has been a significant rise in sexual offenses in Sri Lanka [6,7]. Improving knowledge in areas such as sex, gender, violence prevention, health, well-being, and reproductive health may positively impact rising sexual offenses in Sri Lanka [8,9,10,11]. Currently, only 6% of Sri Lankan students understand contraceptive methods, though targeted interventions could change this [12]. An exploratory study investigated the relationship between sex education and self-poisoning, involving 298 self-poisoning inpatients and 500 controls from Teaching Hospital Peradeniya. About one-third of cases and one-fifth of controls reported no exposure to sexual education [13].

The 2012–2013 National Youth Health Survey found that 50% of post-secondary students in Sri Lanka lacked awareness of sexual and reproductive health (SRH) issues, highlighting an urgent need for change. Comprehensive sexuality education, as seen in Western Europe, shows that effective sex education can be implemented in diverse cultural contexts [14]. Only 1% of rural Sri Lankan students have adequate SRH knowledge [15,16]. Enhancing sex education could greatly improve this, positively impacting adolescents by preventing issues like child sexual abuse [17,18,19,20,21].

In Sri Lanka, sex education is part of the Grade Seven curriculum. However, the supplementary textbook on reproductive health, *Hathe Ape Potha*, has faced opposition from various social groups [22,23,24]. Definitions and guidelines for quality sex education vary widely across countries, cultures, and organizations, underscoring the need for a nuanced approach in diverse settings [25]. According to the World Health Organization (WHO), SRH education provides young people with accurate, age-appropriate information essential for health and survival, fostering awareness of STDs, gender equality, and the prevention of teenage pregnancy, abortion, and sexual abuse [26]. However, concerns from many parents and educators about potentially promoting adolescent sexual behavior highlight the complexity of implementing such programs. Their active participation and careful attention to cultural factors are crucial for the effective delivery of sex education [12,27].

While the importance of sex education is recognized, its implementation for youth remains poorly understood [1]. High rates of rape cases and inadequate sex education suggest a need to explore the complex factors affecting targeted prevention strategies. By examining Sri Lanka’s experience with sex education, researchers can enhance their understanding of how these programs can be adapted in culturally diverse settings, thereby challenging the existing literature. This study explores factors surrounding the inadequacy of sex education and those influencing SRH education quality in Sri Lanka, employing the health belief model (HBM) for analysis [16,22].

### 1.1. Theoretical Perspective

Despite the recognized importance of sex education, its effective implementation for youth remains insufficiently understood [1]. The high rates of sexual offences, coupled with inadequate sex education in Sri Lanka, point to a need for a comprehensive exploration of the factors affecting prevention strategies. Examining Sri Lanka’s experience provides an opportunity for researchers to deepen their understanding of how sex education programs can be tailored to diverse cultural settings, offering insights that may challenge and expand the existing literature on universal education approaches.

This study specifically investigates factors contributing to the inadequacy of sex education and influences on the quality of sexual and reproductive health (SRH) education in Sri Lanka. Using the health belief model (HBM) as a theoretical framework, this study aims to assess perceptions and motivations surrounding SRH education. By analyzing the unique socio-cultural and structural barriers within Sri Lanka, researchers can provide recommendations for effective program adaptation that considers the country’s distinct cultural dynamics. Ultimately, this study seeks to contribute to a nuanced understanding of how tailored sex education can address specific risks and enhance SRH outcomes for youth in diverse settings [16,22].

### 1.2. Literature Review

Despite a large amount of literature on the perceived benefits of quality SRH education and the HBM factors influencing SRH education separately, the relationship between the two has received scant attention. As stated previously, the prevalence of contraception use and the level of sex education awareness have been extensively reported in numerous research studies. However, there has not been much research into the factors that influence the efficacy of sex education, especially in Sri Lanka. On this basis, several knowledge gaps have been identified in the literature regarding the factors affecting the efficacy of sex education. Thus, based on the literature review, the following hypotheses were built to close the knowledge gap. These findings are crucial for understanding the effectiveness of sex education and have the potential to significantly impact the development of future policies and practices in this field.

### 1.3. External Cues to Action

External cues to action, as defined in the health belief model (HBM), refers to the prompts and reminders from the external environment that can influence one’s preparedness to act [28]. In the context of SRH education, these cues can be incorporated by offering prompts and reminders to promote safe behavior. A significant part of these cues are media campaigns, which play a crucial role in informing and promoting safe behavior. For instance, campaigns stressing the value of wearing protection, signs, or reminders from classmates or teachers can all serve as external cues. These cues play a crucial role in translating intentions and knowledge into actual behavior [29].

The term, “cues to action”, pertains to the notion found in the health belief model (HBM) and denotes the catalysts that induce people to undertake behavior relevant to their health. Although the precise context and health behavior being investigated will determine the variables to be measured, standard variables for “cues to action” measurement include internal, external, situational, and cognitive cues [29]. There are two primary categories of external cues: negative social impacts and media and informational cues. Cultural norms can have a significant impact on SRH education, underscoring the need for cultural sensitivity in our work.

Events or information from the outside world can serve as external cues to take action. These cues, known as ‘external cues to action’, are cultural norms: accepted ideas, attitudes, and customs within a group that shape individual behavior. Cultural norms provide an operational definition of “External Cues to Action” for this study. This paper aims to explain and expand upon this operational description, elucidating how cultural norms function as extrinsic stimulants that affect people’s perceptions of SRH schooling.

Several studies have shown how youth have felt pressured by external cues to action, and how SRH policies and sexuality education were based on social status and cultural values [30,31,32]. External cues related to SRH might be found in societal norms and cultural behaviors. These norms have the power to help or impede people’s access to resources and information, which can impact their choices and actions [33].

Programs for peer-led education use the power of social networks to encourage safe sexual behavior [34]. Social norms in UK university male social circles have a negative impact on how people perceive and understand sexual information, in contrast to the traditional culture and environment of Palestine [33]. Culture has been a persistent barrier in Mexico because it is prohibited in families, and there has been opposition to sex education [35]. However, comprehensive sex education is a powerful tool that can counteract these negative cultural norms. SRH knowledge is essential to reduce teenage pregnancy in Sri Lanka as it helps young adults make the right decisions on sexual health and practices [16]. It is evident that culture has a negative impact on young people’s perceptions of sex education. This effect is further exacerbated by the absence of comprehensive and inclusive sex education programs in these countries. Without the right direction and education, young people might rely on false beliefs and damaging stereotypes that are supported by their cultural norms. Therefore, leveraging external cues to put in place comprehensive sex education programs becomes essential in fostering positive attitudes toward sexuality in young people. Thus, the first hypothesis is that these findings can inspire and guide the development of more effective sex education programs, policies, and practices, particularly in Sri Lanka.

**H1.**  *External Cues to Action significantly impact the Perceived benefits of quality SRH education.*

### 1.4. Self-Efficacy

The confidence in one’s capacity to carry out the suggested action successfully is known as self-efficacy [28]. Increasing students’ self-efficacy is crucial to giving them the confidence to make safe sexual decisions. Skill-building exercises, including proper condom usage, effective communication about sex and limits with partners, and accessing sexual health services, should be a part of sex education. For instance, role-playing scenarios, where students practice condom usage or communication with a partner, can be effective. Similarly, interactive techniques, such as group discussions or peer-to-peer teaching, can also enhance self-efficacy [29].

This study’s operational definition of self-efficacy is the degree of embarrassment while discussing or interacting with SRH topics. Educators must understand that the degree to which people feel they can confidently and efficiently engage in SRH-related conversations and activities without feeling uncomfortable or ashamed is reflected in this operational definition. Lower levels of embarrassment are associated with higher levels of self-efficacy, indicating that people feel more confident and at ease when handling SRH concerns. On the other hand, increased embarrassment indicates reduced self-efficacy and can impede candid communication and learning in SRH educational environments. Educators play a pivotal role in improving self-efficacy and the efficacy of SRH education programs by addressing and understanding the factors that lead to embarrassment. This will also encourage more positive health outcomes.

It has been shown that teenagers’ sense of self-efficacy is worse when they feel embarrassed to take SRH courses. In Malaysia, teenage self-efficacy for safer sexual behaviors is influenced by internet-based program [36]. A meta-analysis was conducted by a group of researchers to evaluate the efficacy of SRH programs in India, with a focus on self-efficacy as a critical outcome measure. They have identified that peer education would improve adolescent self-efficacy [37].

The study found that a proper sex education program, especially a teacher-led one, increases positive attitudes in teachers and students, builds confidence to provide sex education, and makes the environment more comfortable, implying the need for a precise program for students and teachers [38]. Fear, embarrassment, discomfort, and humiliation held back child–parent communication about SRH matters [39]. Teachers were uncomfortable in the classroom when teaching sex education [40]. Young adults expressed the stigmas in condom purchasing, carrying, or requesting, and were embarrassed to inform their significant partners about sexually transmitted infections (STIs) [41,42,43]. Undergraduates in the UK were embarrassed to visit a sexual health clinic for guidance due to associated stigmas [42]. University and school sex education is inadequate for young people, especially in industrialized nations like the UK and USA. Developing countries like Sri Lanka should evaluate the issue and strengthen sex education programs to reduce misconceptions and fear. These reveal that undergraduates, parents, and teachers were afraid and embarrassed to discuss SRH matters to educate students and explore how parental communication influences adolescent self-efficacy for safer sex practices. Therefore, the second proposed hypothesis is developed, as the urgency and relevance of this research topic cannot be overstated; it directly impacts the health and well-being of young people, particularly in developing countries like Sri Lanka.

**H2.**  *Self-Efficacy significantly impacts the Perceive benefits of quality SRH education.*

### 1.5. Perceived Barriers

Perceived barriers are a crucial aspect of our study. The perceived barriers refer to the notion that the suggested course of action will have both material and psychological costs [28]. It is of utmost importance to recognize and remove these obstacles for safe sexual behavior. These barriers, which may include ignorance about sexual health, shame over purchasing condoms, or lack of access to contraception, significantly impact the perceived benefits of quality sexual and reproductive health education. Addressing these barriers is essential for the effectiveness of sexual and reproductive health education. Strategies for overcoming these barriers, such as educating people in bargaining techniques, expanding access to contraception, and busting myths, should be a cornerstone of effective sex education [29].

On the other hand, perceived benefits are the positive outcomes that individuals believe will result from adopting recommended behaviors. These benefits, such as reduced risk of health problems, play a significant role in motivating individuals to engage in safe sexual practices. For instance, sex education that emphasizes the advantages of condom use, abstinence, and mutual monogamy can influence students to adopt these practices as they perceive them to be effective in preventing unfavorable health outcomes [29].

Perceived barriers are operationalized for this study based on people’s views. These viewpoints cover various opinions, from acceptance and openness to opposition or even skepticism. Comprehending these viewpoints is essential since they directly affect how SRH education is viewed, accepted, and used in communities and educational settings. Several things might give rise to perceived hurdles, including cultural standards, religious convictions, misunderstandings about the subject matter or goals of SRH education, apprehension about controversy or social criticism, and worries about the morality or age appropriateness of the material. To address these alleged obstacles, offering correct information and creating a welcoming atmosphere is necessary. This welcoming atmosphere promotes communication and tolerance for differing viewpoints making the audience feel accepted and included. This will eventually advance inclusive and thorough SRH education for everyone. Teachers’ perceptions are obstacles to providing sexual health education in the classroom, such as worries about the reactions of parents and curricular limitations [10]. According to a different study, perceived obstacles, such as a lack of resources and opposition from school officials, prevent comprehensive sexual health education from being implemented in secondary schools [12].

Many studies worldwide have identified that perceived barriers cause a negative attitude toward SRH education, while perceived benefits create positive attitudes. Many researchers revealed that students had positive attitudes toward SRH education and normalizing SRH education as a part of learning systems [43,44]. A systematic review examined perceived barriers to sexual health education and HIV prevention among African American women, including stigma and mistrust of healthcare providers [45]. It is crucial to provide reliable information access and communication facilities for female students regarding SRH concerns, as this is an area that needs immediate attention [46]. Sex education and sex have been identified as taboo or stigmatic matters to discuss by most people [43,47].

Moreover, several studies have identified that sexual violence can be prevented or eliminated by providing SRH education, but undergraduates have been shown to lack participation in those programs [47,48]. Most immigrant parents in foreign countries have negative thoughts and opinions regarding sex education of their children [47]. A South African study implied that male students had negative attitudes towards condoms as a contraceptive, and they refused to use condoms for various reasons [41]. The lack of education they have had regarding contraceptives is a significant concern of SRH education. Unlike most of the parental points of view, some studies have shown that some parents were not ready to accept the usage of contraceptives since they prefer abstinence methods. Those parents had negative attitudes toward sex education [49]. China, an Asian country with a similar culture to Sri Lanka, identified that male and female undergraduates had different attitudes toward sex. Male students were more into risky sexual behaviors, and female undergraduates were less likely to accept premarital sex, but female undergraduates had positive attitudes towards homosexuality [50]. Parents have contradictory views on sex education and the degree program area’s effect on the knowledge level of sex education among undergraduates. At the same time, differences in attitudes by gender were especially stressed. UNESCO’s report discusses global challenges and perceived barriers to implementing comprehensive sex education, including cultural resistance and political constraints [21]. Perceived barriers related to adolescent health literacy and an understanding of reproductive health focus on individual and parental negative attitudes towards sex education [51]. This evidence shows that knowledge and information on SRH education were not evenly distributed due to perceived barriers. These barriers can vary widely and may include cultural norms, lack of resources, social stigma, and institutional constraints. It is crucial to address this uneven distribution and ensure equity in SRH education. The potential negative consequences of not addressing these barriers are significant, underscoring the urgency and importance of our research [51]. Thus, the third hypothesis is developed as follows:

**H3.**  *Perceived Barriers significantly impact the perceived benefits of quality SRH education.*

### 1.6. Perceived Susceptibility

Perceived susceptibility, a pivotal concept in our study, is the individual’s perception of their likelihood of experiencing adverse sexual health outcomes, such as STIs or unplanned pregnancies [28]. In the realm of sexual education, realizing students’ perceived susceptibility is crucial, as it enables the customization of educational interventions to their unique needs. For example, if students underestimate the risks of STIs, they may be less inclined to adopt preventive measures [29].

For this study, perceived susceptibility has been operationalized as people’s knowledge and comprehension of SRH education about their vulnerability. This idea is implemented by measuring people’s understanding of how vulnerable they are to dangers and difficulties connected to SRH. Designing successful sex education programs that address students’ concerns and encourage informed decision-making requires an understanding of perceived vulnerability [52]. Parental reports of sexual communication with children across grade levels, investigating how perceived susceptibility affects parent–child discussions about sexual health—the effects of an intervention aimed at encouraging international health education recommendations among German high school students [53]. According to another study, students’ health-related knowledge and behaviors have significantly improved. Along with embracing healthier lifestyle choices, such as more frequent exercise and better eating habits, participants showed higher awareness of issues related to nutrition, physical activity, and mental health [54]. Another study discussed the advantages of sexual expression for older persons’ health, addressing perceived lifetime susceptibility to sexual health problems [55].

While human immunodeficiency virus/acquired immunodeficiency syndrome (HIV/AIDS) awareness was relatively high among Chinese undergraduates majoring in medicine, the research also identified a key area for improvement. Most undergraduates were aware of contraception [56,57], but the understanding of STIs among young adults and undergraduates could have been better [58,59,60]. This finding underscores the need for future research and policy development in this area. Many studies have highlighted the need for perceived susceptibility among undergraduates. For instance, female undergraduates in Brazil had not used contraceptives due to a lack of awareness of unintended pregnancy [61]. Similarly, undergraduates in Italy had poor sexual health knowledge of contraception [62]. However, Korean female undergraduates had more fertility knowledge, and male undergraduates had more understanding of contraceptives [63]. The research also highlighted that unplanned, teenage, and non-marital pregnancies were due to a lack of contraceptive knowledge among Sri Lankan women [64]. These findings underscore the importance of addressing these perceptions to promote effective sexual health education and behavior change. Thus, the fourth hypothesis is developed as follows:

**H4.**  *Perceived Susceptibility significantly impacts the perceived benefits of quality SRH education.*

### 1.7. Perceived Severity

Perceived severity, the belief that the consequences of risky sexual behavior are significant, is a key concept in sexual health education. To promote preventive behaviors, educators should provide comprehensive sex education that covers the physical, mental, and social effects of STIs and unplanned pregnancies. This holistic approach ensures that students have a thorough understanding of the risks involved, thereby increasing their perceived severity [29].

One way to put perceived severity into action is by evaluating the level of institutional support. The extent to which educational institutions and other organizations address SRH issues—such as sexual and reproductive health awareness, STI prevention, and reproductive rights—is a clear indicator of this support. Schools that prioritize SRH education demonstrate their commitment to addressing the seriousness of SRH-related issues that people may encounter, such as the health risks and social consequences individuals may face. This support often includes integrating the curriculum, funding programs and resources, training educators, and implementing policies that promote inclusivity and comprehensive SRH education. By supporting SRH education efforts, institutions can effectively communicate the gravity of these challenges and empower individuals to make informed decisions about their health and well-being.

Several critical factors have been identified in studies as influencing the standard of SRH education in Sri Lanka. The absence of assistance from schools and colleges and knowledge gaps between the sexes due to discovering false information sources underscore the need for a collaborative effort [15,65]. Though not currently taught in Sri Lankan schools, sex education is a necessary step [22,66]. Few undergraduates in Sri Lanka engage in risky sexual behavior, are aware of contraceptives, or are likely to engage in unsafe sexual behavior. These facts make it clear that a collective effort is required to address the ignorance of SRH among Sri Lankan undergraduates [67].

The quality of SRH education offered by schools and universities has been deemed inadequate due to limited resources and insufficient support, which has been recognized as an obstacle to providing comprehensive SRH education. The need for a holistic approach is evident, as lack of support from schools was seen in Thailand, where the focus is homosexuality as it primarily focuses on heterosexuality, and most of the textbook contents were outdated, inaccurate, and impractical [68]. To effectively lower the prevalence of both, extensive teaching and intervention programs are required, addressing both the perception of IPV severity and the associated sexual risk behaviors among college students [69]. The psychological aspects of sexual health behaviors are crucial, as the associations among young adults between condom use, the perceived seriousness of STDs, and self-compassion highlight the psychological aspects of sexual health behaviors [70]. The need for mentors, resources, and curriculum regarding SHR education for young male adults in schools was considered a challenge in Iran [71]. The support from universities regarding contraceptives has been discussed deeply in past studies [72,73]. With institutional support, perceived severity and other factors impacting the outcomes of teenage sexual health can be minimized [74]. Media literacy in sex education is highlighted by the perceived severity of sexual media and social media’s influence on teenagers’ sexual development and engagement in risky sexual behaviors [75]. In a study in South Africa, great support from the university was given to undergraduates, but there was a lack of structured programs [76]. A serious teaching methodology for sex education would help students enhance their knowledge of sexuality in daily life [77]. Recommendations for incorporating comprehensive sex education that addresses the perceived seriousness of sexual health issues and encourages healthy behaviors are part of the World Health Organization’s guidelines for school health promotion [78]. A broad study based in Sub-Saharan Africa reviewed the effects of comprehensive sex education taught in schools on teenagers’ hazardous sexual behavior and STI rates, with an emphasis on perceived severity as an outcome measure [79]. Designing sex education programs that successfully communicate the risks and consequences of sexual behaviors requires an understanding of perceived severity. Thus, the last hypothesis is as follows:

**H5.**  *Perceived Severity significantly impacts the perceived benefits of quality SRH education.*

### 1.8. Quality of Sex Education

Various organizations and research have presented the concept of ‘sex education’ in different ways [23,33]. However, the curriculum and syllabus must meet high standards, encompassing essential elements, and ensuring that the content is clear and coherent. This will significantly improve the quality of sexual education offered to students [18]. High school students who receive comprehensive sexual health education experience a positive transformation in their health outcomes. Importantly, those who received comprehensive sex education demonstrated greater sexual health knowledge, increased condom usage, and reduced rates of sexually transmitted infections (STIs) and unintended pregnancies [80,81]. Comprehensive sex education has led to a significant decrease in the pregnancy rate among adolescent females [31]. Implementing evidence-based programs that teach students conflict resolution, problem-solving, and emotional control skills is crucial. These programs aim to create safer school environments and reduce instances of bullying, violence, and injury [32].

The sex education provided to children in Western countries is inadequate and worrisome. Concerns have been raised about the quality of sex education delivered, and previous studies have recommended improvements in this area [82,83]. Previous research on Sri Lankan education has shown that sex education is not taught as a distinct subject. However, studies have found that students recognize the importance of sexual and reproductive health (SRH) knowledge and believe it should be included as a subject in schools [15,65,66]. The authors of a study in an inner-city Cleveland middle school emphasized the critical need for standardizing sex education by ensuring that essential components are consistently taught to students. Their study highlights the urgent need for a uniform approach to sex education to maximize its effectiveness and ensure all students receive comprehensive and accurate information [83]. Nevertheless, the knowledge acquired by students from both their households and schools was insufficient. Past studies have emphasized the need for sex education by highlighting its usefulness and benefits.

### 1.9. Delivery Methods of SRH Education

According to past studies on SRH education techniques and procedures, there are numerous education program approaches based on the subjects highlighted or ignored. The majority of research studies on this issue have recommended teacher-led, educational intervention, internet-based, peer-led, comprehensive, self-study, multimedia model, and abstinence sex education method programs to improve sex education. Teacher-led SRH education is a viable strategy because it allows teachers to deliver more reliable and entertaining information to students [38]. The educational intervention program, counselling, or teaching combines with interventions to improve clinical understanding [83]. An academic service delivered using an online platform is known as internet-based sex education, and because the volume of web users is growing day-by-day, the internet is being used as a mode to publicize SRH and increase sexual knowledge [36]. Peer-led sex education involves learning about SRH from well-trained, motivated, and knowledgeable peers. Sometimes considered as part of comprehensive teaching, SRH attempts can be categorized solely as ‘peer-led SRH education’ that can affect pupils with openness and many other benefits [37]. Comprehensive sexual education covers abstinence, contraception, including emergency contraception, reproductive choice, LGBT and questioning issues, anatomy, development, puberty, relationships, and everything else covered in a standard sexuality education program with traditional teaching methods. CSE can be used as a fruitful SRH strategy with a well-structured curriculum and teaching methodologies to enable students to make better decisions [21]. The self-study website approach is one of the methods that can deliver SRH knowledge by using fewer resources and having less one-to-one contact [54]. Multimedia can help reach youngsters and enhance SRH awareness. Instead of not having SRH awareness programs, it can be effective [84]. A sexual education program with a heavy emphasis on abstinence until marriage is known as an abstinence approach [83].

### 1.10. Conceptual Framework of the HBM

The proposed conceptual framework of the HBM, with its two major components, is of significant importance: the individual’s perceptions about SRH education and the perceived benefits of quality SRH education. These components play a crucial role in shaping our understanding of sexual and reproductive health education.

Individual Perceptions: Individual perceptions are beliefs about how likely someone thinks they are to be affected by sexual abuse and how serious they think it is. In this review, we look at what adolescents (students) believe about their risk of sexual abuse and how quality sexual and reproductive health (SRH) education can help prevent it. If students’ perceptions change because of new knowledge, it can lead to the successful implementation of quality education, thereby reducing the risk of sexual abuse. Based on the HBM concepts, five variables are identified as independent variables. External cues to action, measured through the influence of cultural norms on SRH education, is considered the first variable. The second variable, self-efficacy, was operationalized and measured using embarrassment for SRH education. The third variable is the HBM concept of perceived barriers and perceived benefits; this is measured through the recognized attitude toward SRH education. The fourth variable, perceived susceptibility, is measured by awareness level. The fifth, and final, variable, perceived severity, is measured by institutional support.

**Figure 1:**
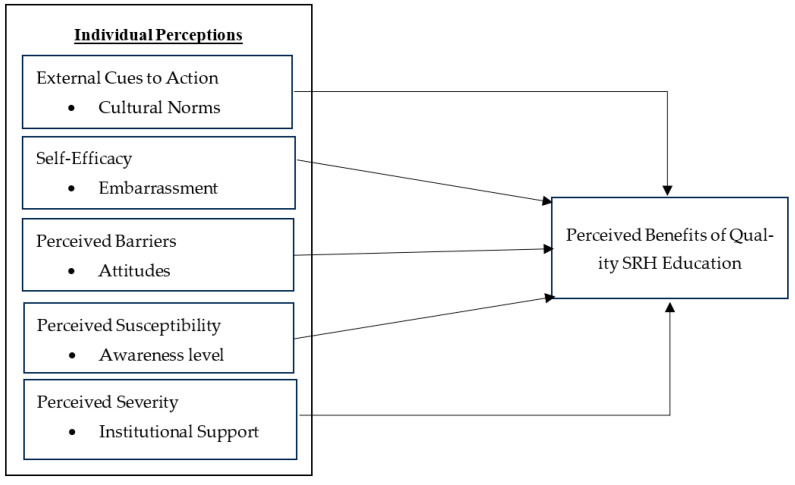
Conceptual Framework of the HBM.

Perceived benefits of quality SRH education: This is considered a dependent variable in the proposed conceptual framework. It refers to how much students believe they benefit from maintaining a high-quality SRH education (Figure 1).

### 1.11. Significance of This Study

Most Western nations are gradually bringing sexual education into the mainstream. Contrarily, despite being problematic, attempts to introduce sexual education are still met with skepticism in most Asian countries. This study significantly contributes to Sri Lankan college students’ efforts to reduce potential social burdens. Few studies examined variables influencing the standard of SRH education, particularly in the Sri Lankan context. Therefore, it is a subject that merits investigation to close the gaps in Sri Lankan sex education. This study aims to pinpoint the variables influencing undergraduate sex education standards based on the HBM theoretical framework. Policymakers and educators can create targeted interventions and strategies to raise the standard of SRH education in Sri Lanka by comprehending these factors. This study will also offer insightful advice for other nations whose SRH education initiatives deal with similar difficulties, thereby informing and guiding their efforts.

## 2. Materials and Methods

This study employs a positivist philosophical framework and a deductive approach to examine how the health belief model (HBM) determinants affect the quality of sexual education. A quantitative research approach is utilized, employing a survey strategy to efficiently gather extensive data from a large population while minimizing costs, thus ensuring the validity and reliability of the findings. This meticulous approach to data collection and analysis enhances the credibility of the research [85].

### 2.1. Participants

In Sri Lanka, a total of 24 non-state universities and higher education institutes have been established under the Universities Act No. 16 of 1978 [86]. This study’s population consists of undergraduate students from these private sector institutions. Using a single population formula with a 95% confidence level, a 5% margin of error, and a standard deviation of 50%, the sample size was calculated to be 384 undergraduate students. This sample size is considered sufficient to represent the undergraduate population in Sri Lanka [87]. Simple random sampling was employed to enhance the representativeness of the sample. A total of 450 questionnaires were distributed, with 414 responses received. Incomplete and biased responses were excluded to ensure data quality and reliability. Additionally, responses from foreign students were omitted, focusing the analysis solely on local undergraduate students to maintain relevance to the study’s objectives. This refined dataset improves the accuracy of findings by reducing potential distortions from non-representative responses.

### 2.2. Procedure

The present cross-sectional investigation was undertaken throughout the period spanning from October 2021 to December 2021. The present study employed two self-administered questionnaires prepared explicitly for data collection. The first questionnaire focused on measuring students’ perception of the quality of SRH education. The questions were designed and refined in accordance with established questionnaires from prior research [36,61,63,73] and grounded in a thorough review of existing literature. The questionnaire was divided into three sections: “Section A”, “Section B”, and “Section C”. Section A consisted of socio-demographic questions, such as gender, ethnicity, religion, degree program, and residence; whereas Section B consisted of 5-point Likert scale questions to measure independent and dependent variables by utilizing a Likert scale with five response options, which have been assigned the following categories: “5 = strongly agree”, “3 = neutral”, “2 = strongly disagree”, and “1 = strongly disagree”. Initially created by Likert in 1932, the Likert scale is widely employed for assessing survey participants’ views [88]. Determining the central tendency for each variable is achieved by using the mean values of the dataset [89].

Section C employed a linear scale question to assess students’ preferred SRH program. The survey included multiple questions, such as, “What type of sex education do respondents prefer to learn?” Based on past literature, the programs listed included a comprehensive sexual education program, a program that allows students to ask anonymous questions, an abstinence-only program, a program with only learning material without a teacher, peer-led programs, and a self-study website or internet-based program. Three categories of preference were identified: scores of 1–3 were considered low, 4–6 were considered medium, and 7–10 were considered high.

A digital survey was designed and disseminated to four hundred and fifty participants to ensure comprehensive understanding. This process yielded a sample size of three hundred and eighty-four individuals. To address the non-response bias issue, the researchers distributed the questionnaire physically to a total of four hundred and fifty employees. Consequently, the resulting sample size amounted to three hundred and sixty individuals. Four hundred and seventeen people, comprising 92.6 percent of the respondents, participated in the poll. However, 33 questionnaires were deemed ineligible for data analysis due to incompletion. As a result, the final sample size for this study consisted of 384 questionnaires, which accounted for 85.3 percent of the total collected. The high retrieval rate of 85 percent is a testament to the inclusivity of the research process, ensuring a diverse range of perspectives is represented in the findings [90].

The second questionnaire was created based on Section C of the first questionnaire to identify the preferences of educators regarding the type of SRH program. Additionally, two demographic questions—gender and faculty—were included to better understand the sample. This questionnaire was distributed among a randomly selected group of 30 university educators from the primary sample. This approach aimed to achieve a better solution to determine the most suitable program to mitigate the risk, considering the perspectives of both students and educators.

### 2.3. Data Triangulation

Denzin identified four types of triangulations in social research during the 1970s: data triangulation, investigator triangulation, theory triangulation, and methodological or method triangulation [91]. In this work, two triangulation methods were utilized: data triangulation and theory triangulation.

Data triangulation, a method that uses multiple data sources to cover various aspects, such as time, location, and individuals, is a powerful tool in research. By corroborating the findings, it helps to verify them, thereby offsetting any deficiencies in the data and enhancing the reliability of the research. By utilizing the advantages of supplementary data, the accuracy and reliability of the outcomes can be improved. The approach has been extensively used in several sectors to improve the accuracy of results and reduce the possibility of incorrect interpretations [91]. The data triangulation involved cross-referencing the students’ responses for section C with the educators’ preferences for the sort of education. Theory triangulation, on the other hand, entails the application of multiple theories or hypotheses to examine a particular situation or occurrence. This involves analyzing an issue or occurrence from numerous perspectives, employing various methodologies. Employing diverse lenses, each with a specific focus, to scrutinize separate facets or viewpoints. Several theories or hypotheses do not have to be the same or compatible; the more they differ, the more likely they are to address separate issues and/or concerns [91]. The empirical findings in this study used triangulation to demonstrate the internal validity of the ideas of BHM.

### 2.4. Variable Measurement and Reliability

To gain access to consistent and accurate data, this study conducts reliability tests to determine whether the questionnaire can provide standardized and high-quality data and information. Consequently, the internal consistency is evaluated using Cronbach’s alpha, a measure of internal consistency and the degree to which a set of queries is interrelated. Table 1 displays Cronbach’s alpha values for all variables exceeding the expected threshold of 0.7, as the key indicates [92]. Therefore, all variables possess a high degree of dependability.

To test the construct validity of the questionnaire, use Pearson’s correlation coefficient against the critical value (0.148) of the pilot survey [93]. The findings show (Table 1) that all the variables have higher correlation coefficient values against the critical value (0.148) at the 382 (*n* = 2) degree of freedom and 0.01 confidence level. Accordingly, obtained correlation coefficient values are 0.812 for the perceived benefits of quality SRH education, 0.352 for external cues to action, −0.171 for self-efficacy, 0.301 for perceived barriers, 0.721 for perceived susceptibility, and 0.805 for perceived severity, showing the construct validity of the instrument. The strong correlations (0.812, 0.805) demonstrate strong construct validity, while moderate correlations (0.352, 0.301) indicate valid, though less strong, relationships. The negative but significant correlation (−0.171) for self-efficacy, despite its direction, also supports construct validity within its theoretical context.

### 2.5. Methods of Data Analysis

The collected primary data has been meticulously analyzed using the robust STATA/SE version 14 software. This software includes a wide range of statistical analysis techniques, such as descriptive statistics and the ordinal probit model. We have also utilized the Word cloud feature to analyze open-ended questions, ensuring a comprehensive analysis of the data. This rigorous approach to data analysis strengthens the validity of our findings.

The mean value and standard deviation of the levels of agreement and disagreement are calculated using descriptive statistics. In a novel approach to our research, we have employed an innovative ordered probit regression model [94]. This model, which simply reflects the ranking of the results, is a unique contribution to the field. The model outputs consist of maximum likelihood. The ordered probit model assesses the impact of independent variables based on the probability of their association with different levels of SRH education quality. The perceived benefits of quality SRH education were measured across three levels, defined as low, moderate, and high, and an ordered probit model was used to extend the study. This model is an extension of the conventional probit model for cases in which a dependent ordinal variable has more than two outcomes [94]. The model’s general specifications are as follows:

(1) $$y_{i}^{*}=\beta x_{i}+e_{i}$$

where *x_i_* = explanatory variables, *β* = unknown parameters, *e_i_* is a random-error term assumed to be normally distributed with zero means, and *y* = the perceived benefits of quality SRH education (latent/dependent variable) and characterized as a discrete variable, with values ranging from 1 to k. This study used a 5-point Likert scale to categorize the perceived benefits of quality SRH education into the following three (k = 3) groups:

Group 1 (*y* = 1): low level is denoted as respondents with less than or equal to two on a 5-point Likert scale ($y_{i}^{*}$ ≤ *μ_1_*).

Group 2 (*y* = 2): moderate level is denoted as respondents with three of 5-point Likert scale (*μ_1_* < $y_{i}^{*}$ ≤ *μ_2_*).

Group 3 (*y* = 3): high level is denoted as respondents with greater than three and less or equal to five on a 5-point Likert scale ($y_{i}^{*}$ > *μ_2_*).

The model we have used can effectively determine the predicted probability and marginal effects for each SRH education category. It is important to note that changes in explanatory variables have a significant impact on the prediction probability of the perceived benefits of quality SRH education in each category group, as demonstrated by the marginal effect. This means that the independent variables we have considered are crucial in determining the quality levels of SRH education. The ordered probit model has allowed us to estimate the marginal effects, which quantitatively measure the influence of explanatory variables on the probability of being in different quality levels of SRH education. The significant variables are chosen using the forward stepwise regression technique, a robust method that ensures the reliability and validity of our findings by selecting the most influential variables in a systematic and unbiased manner (Table 2).

A word cloud has been used to analyze the two open-ended questions. A word cloud is performed to rapidly understand the most frequently appeared terms in a body of text by using a graphical illustration of word frequency and quality [95]. The most frequently appeared terms from Word Cloud represent what the mostly stated respondents’ views regarding sex education in Sri Lanka among UGC-approved non-state undergraduates and ideas about sex education in universities were. Pro Word Cloud in Microsoft Word 2019 has analyzed the open-ended questions mentioned above.

## 3. Results

### 3.1. Demographics

The demographic data gathered for the study sample, as shown in Table 3, is based on a sample of 384 Sri Lankan non-state university students who were randomly chosen from the UGC-approved non-state universities, ensuring a fair and unbiased representation of the student population. The data reveals that 52.3% of the sample’s university students were men, and 47.7% were women. A significant 64.8% of the students who took part were Sinhala, while 52.3% identified as Buddhist, and 23.4% as Christian. Business students comprised 32% of the respondents, while IT, engineering, and faculty students comprised 19%, 18%, and 13.3%, respectively. A total of 46.1% of the participants in this research study were from urban areas, 38.5% were from suburban areas, and the remaining 15.4% were from rural areas. Thus, most of the sample consists of urban males identifying as Buddhist and Sinhala.

### 3.2. Hypothesis Testing

After obtaining the Pseudo R^2^ value from the initial ordered probit model (Table 4), marginal impacts for low, moderate, and high SRH education quality were calculated. The results showed that external cues to action (*p* = 0.878) and perceived barriers (*p* = 0.892) were not statistically significant; while other variables, like perceived susceptibility, perceived severity, and self-efficacy, had significant impacts. These findings inform SRH education policies. These findings carry important implications for shaping SRH education policies and practices.

This study used a stepwise forward technique to derive the final ordered probit model results (Table 5), selecting variables based on statistical significance. The findings indicate a negative association between a lower level of self-efficacy regarding SRH education and the probability of it being rated as “high” quality. A 1% rise in poor self-efficacy reduces the likelihood of high-quality SRH education by 0.06 percentage points, while increasing the probability of it being classified as “low” by 0.10 percentage points. The “moderate” category saw a 0.04 percentage point decline. These results suggest that higher poor self-efficacy reduces the perceived benefits of SRH education quality.

The derived data, as presented in Table 5, demonstrate the positive outcomes of increased awareness. The marginal effects related to the factors show that a 1% increase in perceived susceptibility among university students leads to an approximate 0.07 percentage points increase in the probability of the perceived benefits of quality SRH education being in the “high” category and 0.04 percentage points in the “moderate” category. Conversely, a 1% increase in awareness level results in a decrease of 0.12 percentage points in the probability of the perceived benefits of quality SRH education being in the “low” category. These findings suggest that when the awareness level of undergraduates increases, the perceived benefits of quality SRH education also rise, particularly in the high-level category.

The role of institutional support in enhancing the perceived benefits of quality SRH education is not just crucial but also promising, as evidenced by the data in Table 5. A mere 1% increase in the proportion of support from the university/school is associated with a significant decrease of 0.50 percentage points in the probability of the perceived benefits of quality SRH education for undergraduates being categorized as “low”. Similarly, a 1% increase in the proportion of support for SRH education from the institution is associated with a promising increase of 0.32 percentage points in the probability of the perceived benefits of quality SRH education reaching a “high” level. These findings underscore the potential for improvement in SRH education with institutional support.

### 3.3. Word Cloud Analysis for Open-Ended Questions

**Figure 2:**
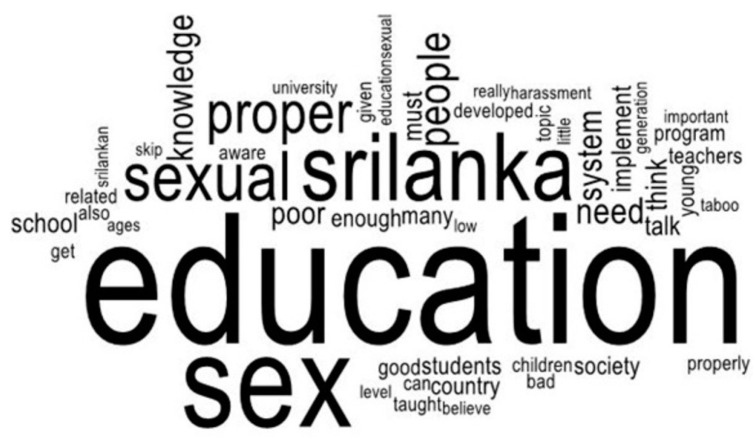
Types of sexual education.

Figure 2 underscores the importance of a well-organized approach, as it shows the most common keywords when answering the question, ”What do you think of sexual education in Sri Lanka?” The most common keywords include the following: ‘people’, ‘Sri Lanka’, ‘think’, ‘knowledge’, ‘must need’, ‘proper’, ‘system’, ‘sexual’, ‘sex’, ‘education’, ‘university’, ‘skip’, ‘poor’, ‘low’, ‘taboo’, ‘bad’, ‘program’, etc. According to Word Cloud’s relative frequency of words, most respondents stressed the need for a systematic sexual education program in Sri Lanka, using keywords that highlight the importance of a well-organized approach. This figure highlights the keywords of respondents’ answers, supporting the necessity for structured sex education in society, as recommended in earlier literature.

**Figure 3:**
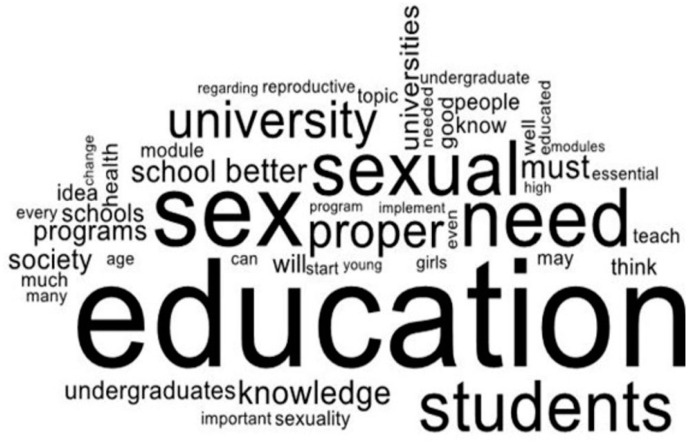
Perspective of proper SRH education.

Figure 3 best depicts undergraduates’ views on university SRH education. ‘University’, ‘need’, ‘proper’, ‘sex’, ‘education’, ‘module’, ‘knowledge’, ‘better’, among others, are the most commonly used keywords when answering the question, “What are your ideas about proper sexual education as an undergraduate and your idea about sexual education for university students in Sri Lanka?” This figure showed that universities require sex education modules or programs. Past university student studies have shown the frequency of the words by stating and emphasizing almost the same aspect of the issue and solid literature on sex education issues.

### 3.4. Triangulation

#### Most Preferable SRH Program Based on the Students’ and Educators Point of View

In section C of the main questionnaire, the undergraduates were allowed to select a sex education program of their preference, and the researchers provided them with six options that were supported.

In Section C of the main questionnaire, undergraduates were asked to choose a preferred sex education program from six options provided by the researchers. Table 6 shows that among the 30 participants, 10% were from the arts faculty, 33.3% from business, 23.3% from engineering, 10% from humanities, 16.7% from IT, and 6.7% from science. According to Table 6, 60% of educators favored a comprehensive sex education program, while only 3.3% opted for a program allowing students to ask questions anonymously. The remaining preferences included programs without a teacher (23.3%), learning materials only (6.7%), peer-led programs (6.7%), and self-study or internet-based programs (6.7%). Notably, no educator supported an abstinence-only program. Gender-wise, 72.2% of female educators preferred comprehensive sex education, while all male educators suggested a program without a teacher, relying solely on learning materials.

Considering Table 7, a significant 56% of undergraduates have chosen a comprehensive sexual education program for their universities, while 27.3%, 4.4%, 3.1%, 3.9%, and 5% of the total sample rated a program that allows students to ask anonymous questions, an abstinence-only program, a program with only learning material without a teacher, a peer-led program, and a self-study website or internet-based program, respectively.

This study triangulated undergraduate and non-state university instructor outcomes to analyze six SRH education strategies to recommend a program for Sri Lankan undergraduates.

**Figure 4:**
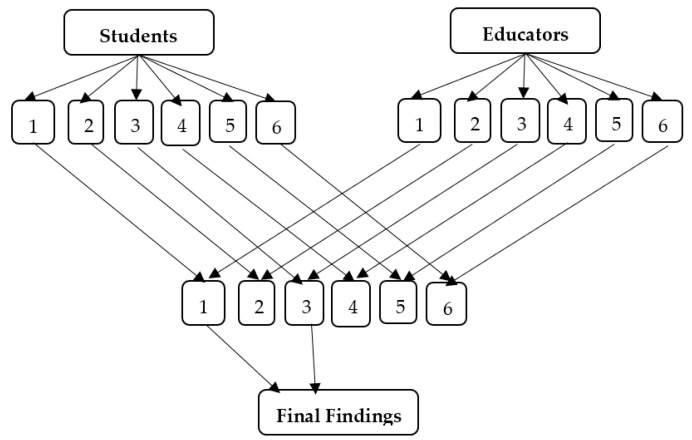
Data Triangulation on Students and Educators Preference.

Figure 4 demonstrates that both parties, students and educators, agreed on comprehensive SRH education and self-study. Most undergraduates (56%) and educators (60%) not only prefer but strongly advocate for a comprehensive SRH education (Table 7). Thus, almost half of undergraduates and instructors chose comprehensive SRH education. An abstinence-only program that educates teens that virginity is the only moral sexual expression was assessed by only 4% of undergraduates. Out of 30 educators, no one has advocated an abstinence-only university sex education program. A sexual education program without a teacher, relying on learning materials (video/audio) or peer-led instruction, was preferred by 3% and 4% of undergraduates, respectively, while 5% favored a self-study website or internet-based program. In total, 12% of undergraduates preferred online and peer-led sex education over teacher-led programs. Similarly, 36.7% of educators recommended sex education without teacher involvement.

## 4. Discussion

Amidst the rising sexual offences in Sri Lanka, the perceived benefits of quality SRH education for students remain a contentious issue. With its unique focus on non-state undergraduates, this study aims to estimate the factors influencing the perceived benefits of quality SRH education in Sri Lanka. This study hypothesizes that the five HBM criteria, external cues to action, self-efficacy, perceived barriers, perceived susceptibility, and perceived severity, may significantly impact its quality. It is crucial to note the potential negative consequences of the lack of comprehensive sexual education in Sri Lanka, which should engage researchers in finding effective solutions.

The first hypothesis asserts that external cues to action significantly influence sexual and reproductive health (SRH) education within the Sri Lankan context. However, the results from a rigorous analysis using an ordered probit model (Table 4) revealed no statistically significant evidence to support this hypothesis. Consequently, it can be concluded that external cues to action do not affect the perceived benefits of quality SRH education in Sri Lanka, leading to the rejection of the hypothesis. This finding challenges the existing understanding of SRH education in the country.

It is important to acknowledge that previous research generally supports the notion that external cues significantly impact SRH education and policy implementation [31,32,33]. This study offers a different perspective, emphasizing the complexity of external cues as a factor influencing SRH education. A lack of comprehensive sexual education among undergraduate students is linked to an increased likelihood of engaging in risky sexual behaviors [33].

In Thailand, the effectiveness of sex education campaigns faces challenges due to socio-cultural, religious, and political factors [68]. Thus, cultural awareness is essential, as traditional societal norms shape the understanding of sexuality. While Sri Lanka shares cultural norms with ASEAN countries, these norms do not significantly influence the perceived benefits of quality SRH education in Sri Lanka.

This finding aligns with Henok and Takele [45], who argued that cultural values and beliefs can impede sexual and reproductive health (SRH) education. In Sri Lanka, traditional and conservative views hinder open discussions on topics such as virginity, SRH, and premarital intercourse, rendering them taboo. Consequently, many individuals may lack exposure to external cues like public health campaigns or media messages due to inadequate dissemination or targeting of information. This lack of awareness creates a disconnect between intended external cues and the population’s understanding, limiting their potential influence in Sri Lanka.

Additionally, individual beliefs, experiences, and family influences likely shape perceptions of SRH education more significantly than external cues. People often depend on personal networks and experiences rather than external messages to form their attitudes toward health education [10]. This limited communication obstructs the transmission of beliefs regarding these topics. Furthermore, adolescents are navigating the transition from highly traditional cultures to modern societal norms, as evidenced by their attitudes toward issues like rape and harassment. This context may explain the current study’s finding that external cues to action do not predict the perceived benefits of quality SRH education in Sri Lanka.

The second hypothesis posited that higher self-efficacy is associated with increased engagement in preventive health behaviors and resilience. Given a *p*-value of 0.007, H2 is accepted, indicating that self-efficacy positively influences the perceived benefits of quality SRH education in Sri Lanka. This finding prompts an examination of the quality of SRH education young people receive and highlights the need to enhance self-efficacy among undergraduates for effective SRH education [38]. Previous studies have identified fear of judgment, embarrassment when discussing sexual concerns, and humiliation as barriers to accessing quality sex education. Self-efficacy beliefs significantly impact the initiation and maintenance of health-related behaviors, such as safe sex practices [51,71]. Many individuals avoid discussing sexual health due to feelings of humiliation or discomfort. For example, parents may hesitate to address sex-related issues with their children, while young adults often fear that their inquiries will be misinterpreted [39]. Additionally, undergraduates frequently feel embarrassed to seek assistance from sexual health services [40].

Hypothesis 3 posited that perceived barriers significantly impact the perceived benefits of quality sexual and reproductive health (SRH) education, drawing on insights from previous studies. However, the results of this study indicated that the relationship between perceived barriers and the perceived benefits of quality SRH education was statistically insignificant, with a *p*-value of 0.892, suggesting that perceived barriers do not play a crucial role in predicting the quality of SRH education. This finding contrasts with previous research, which has highlighted negative attitudes toward aspects of sex education among undergraduates in countries like New Zealand, where sex education is often viewed as taboo [43,47]. Similarly, studies from the USA, Sweden, and China revealed varying attitudes toward sex education, particularly among Chinese undergraduates [61]. The discrepancies in findings may be attributed to cultural differences, as these studies were conducted in contexts where sex education is mandatory from an early age. In Sri Lanka, perceived barriers to SRH education are influenced by cultural taboos [11,24], limited awareness of available resources [10,24], and strong familial and societal influences [10]. These factors prioritize personal beliefs over external messages, diminishing the perceived importance of barriers and, consequently, affecting engagement and the perceived benefits of SRH education.

Hypothesis 4, constructed around perceived susceptibility, significantly influences the perceived benefits of quality SRH education. Perceived susceptibility refers to an individual’s assessment of their risk of receiving the worst-case scenario due to a lack of SRH education. This study’s findings match the theoretical concept that higher perceived susceptibility is associated with greater engagement in preventive sex-related issues. If there is a quality SRH education, students’ perceived susceptibility will be enhanced.

This study’s results, indicated by a *p*-value of 0.021 (Table 4), confirm the acceptance of H4, suggesting that enhancing parents’ comfort and knowledge in discussing sexual health can improve perceived susceptibility [53]. This contrasts with findings from Chinese and Korean undergraduates, who showed higher awareness of contraceptives and fertility [59,65]. Previous research consistently highlights that increased awareness among undergraduates enhances the effectiveness of sex education. Many young adults and undergraduates lack an understanding of sexually transmitted infections (STIs) and sexual and reproductive health (SRH) topics [58,59,60]. Similarly, Italian undergraduates demonstrated insufficient knowledge about contraceptives [62]. To enhance sexual health among Brazilian undergraduates, implementing learner-centered interventions is essential [61]. Low awareness levels negatively impact the quality of sex education; thus, increased awareness can lead to improvements in this area. Management support can boost knowledge of diseases like HIV/AIDS and STIs [62]. An information campaign could effectively address access barriers for those seeking information [64]. Furthermore, a *p*-value of 0.000 for hypothesis 5 indicates that perceived severity significantly impacts the perceived benefits of quality SRH education, motivating individuals to heed health warnings.

A *p*-value of 0.000 (Table 4) indicates that support from schools and universities significantly impacts the perceived benefits of quality SRH education, leading to the acceptance of H5. Table 5 illustrates that for every 1% increase in funding from educational institutions, there is a 32% rise in the likelihood of achieving “high” quality sex education. This finding underscores the crucial role of school support, particularly given the challenges posed by outdated curricula and insufficient resources [68]. There is also a documented need for mentors and additional resources in schools to enhance SRH education [71]. This gap highlights the urgent need for action, especially since many universities do not offer courses on contraception or SRH topics [72,73]. Overall, this study emphasizes the importance of institutional support in ensuring that undergraduates receive high-quality SRH education.

Qualitative findings indicate that both students and instructors prefer comprehensive, internet-based sexual and reproductive health (SRH) education. These preferences are supported by previous studies in China, the USA, and Scotland, which advocate for a comprehensive sexual education curriculum that includes topics such as abstinence, contraception (including emergency contraception), reproductive choices, anatomy, development, puberty, and relationships—all typically covered in standard sexuality education programs using traditional teaching methods. This research specifically examines how comprehensive sexual education programs can improve university students’ attitudes and awareness [83]. This study rejects abstinence-only sex education, aligning with earlier research that questions its practicality and effectiveness [83]. While internet-based and peer-led programs can enhance SRH knowledge and alleviate discomfort with face-to-face instruction, they raise concerns about the quality of information due to a lack of instructor guidance. UNESCO emphasizes that comprehensive sex education should involve a structured curriculum and active teaching methods to empower students to make informed decisions [21]. In Thailand, teacher-led programs have been shown to boost educators’ confidence and attitudes toward sex education, highlighting the essential role of instructor involvement [38]. Consequently, sex education programs that do not include teachers are not recommended due to the potential for lower knowledge quality.

The findings revealed that key elements of the HBM, such as self-efficacy, perceived susceptibility, and perceived severity, are essential for improving the effectiveness of SRH programs. These factors drive motivation, participation, and behavioral changes, thereby enhancing the overall quality and impact of SRH education. Therefore, this study demonstrates that the HBM is a valuable framework for understanding the factors influencing SRH education and highlights its significance in shaping effective and impactful programs.

The absence of comprehensive SRH education in Sri Lankan institutions can lead to higher rates of STDs, unintended pregnancies, and the perpetuation of cultural stigma and gender stereotypes. To address these issues, policymakers and educators must prioritize developing a standardized SRH curriculum tailored to Sri Lanka’s cultural context. Strengthening educators’ self-efficacy, launching empowerment programs, and implementing targeted awareness campaigns can enhance students’ understanding and confidence regarding SRH risks. An age-appropriate curriculum, supported by strong school policies and community involvement, will foster greater engagement, awareness, and proactive behavior in students regarding their sexual and reproductive health.

This study has limitations in demographic diversity, potentially introducing bias toward specific cultural, geographical, gender, and sexual orientation groups. Future research should include a broader range of participants, incorporating diverse cultural, religious, educational, and socioeconomic backgrounds. Expanding studies in other regions or countries could provide a deeper understanding of the topic. Additionally, qualitative methods, such as interviews or focus groups, can offer more comprehensive insights into participants’ attitudes, addressing these limitations and enriching the findings.

## 5. Conclusions

In conclusion, the findings highlight the critical roles of self-efficacy, perceived susceptibility, and perceived severity in shaping the perceived benefits of quality SRH education for undergraduate students. Additionally, this study reveals strong agreement between students and educators regarding the need for comprehensive SRH education and the value of self-study, emphasizing the importance of structured, well-supported programs. These results stress the need for standardized SRH education and targeted awareness campaigns to enhance student engagement. Moreover, addressing negative attitudes, such as stigma and discomfort surrounding SRH topics, is essential for improving the quality and effectiveness of SRH education.

## Figures and Tables

**Table 1:** Reliability Statistics of Variables.

Variables	Observations	Cronbach’s Alpha (α)	Correlation Coefficient (r)	No of Items
External Cues to Action	384	0.759	0.352	05
Self-Efficacy	384	0.944	−0.171	05
Perceived Barriers	384	0.816	0.301	05
Perceived Susceptibility	384	0.845	0.721	06
Perceived Severity	384	0.936	0.805	05
Perceive benefits of quality SRH education	384	0.956	0.812	06

Source: Authors’ representation.

**Table 2:** Possible explanatory variables for perceived benefits of quality SRH education.

Variables	Label	Expected Signs
External Cues to Action	1—low, 2—moderate, 3—high	(−)
Self-Efficacy	1—low, 2—moderate, 3—high	(−)
Perceived Barriers	1—low, 2—moderate, 3—high	(+)
Perceived Susceptibility	1—low, 2—moderate, 3—high	(+)
Perceived Severity	1—low, 2—moderate, 3—high	(+)

Source: Authors’ representation.

**Table 3:** Demographic profile of the respondents.

Demographic Factors		Number	Percentage
Gender	Male	201	52.3
	Female	183	47.7
Ethnicity	Sinhala	249	64.8
	Tamil	75	19.5
	Muslim	32	8.3
	Burgher	26	6.8
	Other	2	0.5
Religion	Buddhist	201	52.3
	Christian	90	23.4
	Hindu	51	13.3
	Islam	35	9.1
	Other	7	1.8
Degree program	Arts	51	13.3
	Business	123	32
	Engineering	69	18
	Humanities	25	6.5
	IT	73	19
	Science	29	7.9
	Other	14	3.7
Residence	Urban	177	46.1
	Rural	59	15.4
	Sub-urban	148	38.5

Source: Authors’ representation.

**Table 4:** Initial ordered probit model results.

Variables	Coef.	Std. Err.	z	*p*	95% Confidence Interval	
External Cues to Action	0.022	0.143	0.15	0.878	−0.259	0.303
Self-Efficacy	−0.259 **	0.097	−2.69	0.007	−0.449	−0.070
Perceived Barriers	−0.037	0.274	−0.14	0.892	−0.575	0.501
Perceived Susceptibility	0.296 *	0.129	2.30	0.021	0.044	0.548
Perceived Severity	1.294 ***	0.121	10.71	0.000	1.057	1.531
Pseudo R^2^	0.4032					
Log-likelihood	−215.8464					
No. of observations	384					

Note: * significant at the 5% level, ** significant at the 10% level, and *** significant at the 1% level. Source: Authors’ representation.

**Table 5:** The final ordered probit model results with forward stepwise technique.

Variables	Marginal Effects (in Percentage Points)				
	Estimate	Robust SE	Low (y = 1)	Moderate (y = 2)	High (y = 3)
Self-Efficacy	−0.2594 **	0.2594	0.100 **	−0.037 **	−0.063 **
Perceived Susceptibility	0.2961 *	0.1355	−0.115 *	0.043 *	0.073 *
Perceived Severity	1.2941 ***	1.2941	−0.502 ***	0.186 ***	0.317 ***
Pseudo R^2^	0.4032				
Log-likelihood	−215.8619				
No. of observations	384				

Note: * significant at the 5% level, ** significant at the 10% level, and *** significant at the 1% level. Source: Authors’ representation.

**Table 6:** Educators’ Preference.

Demographic Factors	Type of Method (Percentages)					
	Comprehensive Sex Education Program	A Program that Allows Students to Ask Anonymous Questions	A Program Without a Teacher, with Only Learning Material	A Peer-Led Program	A Self-Study Website or Internet-Based Program	Total
	(60%)	(3.3%)	(23.3%)	(6.7%)	(6.7%)	
**Gender**						
Male	27.8	100.0	28.6	50.0	50.0	33.3
Female	72.2	0.0	71.4	50.0	50.0	66.7
**Faculty**						
Arts	0.0	100.0	14.3	50.0	0.0	10.0
Business	38.9	0.0	28.6	0.0	50.0	33.3
Engineering	27.8	0.0	0.0	50.0	50.0	23.3
Humanities	11.1	0.0	14.3	0.0	0.0	10.0
IT	16.7	0.0	28.6	0.0	0.0	16.7
Science	5.6	0.0	14.3	0.0	0.0	6.7

Source: Authors’ representation.

**Table 7:** Students and Educators Preference.

Serial No.	Type of Method	Students		Educators	
		Preference	Rank	Preference	Rank
1	A comprehensive SRH education program	56	1	60	1
2	A program that allows students to ask anonymous questions	27	2	3	5
3	A self-study website or internet-based program	5	3	8	3
4	Abstinence-only program	4	4	0	6
5	Peer-led program	4	5	6	4
6	Program with only learning material without a teacher	3	6	23	2

Source: Authors’ representation.

## Data Availability

The supporting data set is openly available on Harvard Dataverse at https://dataverse.harvard.edu/dataset.xhtml?persistentId=doi:10.7910/DVN/PS1JES (accessed on 11 November 2024). To protect data, personal information, and responses were kept strictly confidential. Identifiable information was anonymized to ensure participants’ privacy was maintained. Data was stored securely in password-protected files; only the research team could access this information.
